# Retraction Note to: The circRNA circAGFG1 acts as a sponge of miR-195-5p to promote triple-negative breast cancer progression through regulating CCNE1 expression

**DOI:** 10.1186/s12943-022-01613-w

**Published:** 2022-07-08

**Authors:** Rui Yang, Lei Xing, Xiaying Zheng, Yan Sun, Xiaosong Wang, Junxia Chen

**Affiliations:** 1 Department of Cell Biology and Genetics, Chongqing Medical University, #1 Yixueyuan Road, Chongqing, 400016 China; 2 Department of Endocrine and Breast Surgery, The First Affiliated Hospital of Chongqing Medical University, #1 Yixueyuan Road, Chongqing, 400016 China

**Retraction note to: Mol Cancer 18, 4 (2019)**

**https://doi.org/10.1186/s12943-018-0933-7**

The Editor-in-Chief has retracted this article. After publication, concerns were raised regarding similarities in cell and tissue images. Specifically:

- Fig. 3e MDA-MB-231 circAGFG1 and Fig. 8a MDA-MB-231 circAGFG1 + miR-195-5p images appear to contain overlap
- Various panels appear to overlap in Figs. 4c and 8b, with different rotation and brightness/contrast of the images.
- In Fig. 5 g, the mock group images representing CCNE1 and pRB appear highly similar,

Additionally, the authors could not provide ethics approval numbers for the human and animal parts of the study upon request. The Editor-in-Chief therefore no longer has confidence in the presented data.

Junxia Chen has not explicitly stated whether they agree to this retraction notice. Rui Yang, Lei Xing, Xiaying Zheng, Yan Sun and Xiaosong Wang have not responded to any correspondence from the editor or publisher about this retraction.

